# Effect on Reduction in Inflammatory Fluid and Improvement of Cell Membrane/Skeletal Muscle by Far-Infrared Rays Emitted from Loess Bio-Balls During Sleep

**DOI:** 10.3390/biomedicines13071603

**Published:** 2025-06-30

**Authors:** Yong Il Shin, Min Seok Kim, Yeong Ae Yang, Yun Jeong Lee, Gye Rok Jeon, Jae Ho Kim, Yeon Jin Choi, Woo Cheol Choi, Jae Hyung Kim

**Affiliations:** 1 Department of Rehabilitation Medicine, School of Medicine, Pusan National University, Yangsan 50612, Republic of Korea; rmshin@pusan.ac.kr; 2 Monash Health, Melbourne, VIC 3800, Australia; minseok.kim@monashhealth.org; 3 Department of Occupational Therapy, Inje University, Gimhae 50834, Republic of Korea; otyya62@inje.ac.kr (Y.A.Y.); wmfghi69@naver.com (Y.J.L.); 4 R&D Center, eXsolit, Yangsan 50611, Republic of Korea; grjeon@pusan.ac.kr (G.R.J.); jhkim@pusan.ac.kr (J.H.K.); 5 R&D Center, Hanwool Bio, Yangsan 50516, Republic of Korea; hbio1004@naver.com (Y.J.C.); lih1769@naver.com (W.C.C.)

**Keywords:** loess bio-balls, far infrared rays (FIR), lymphatic circulation, inflammatory fluids, bio electrical impedance

## Abstract

**Background**: Far infrared rays (FIR) can promote microcirculation of blood in capillaries and reduce inflammation and edema by improving lymphatic circulation. Although the short-term therapeutic use of FIR is increasingly common in clinical settings, its effects on inflammatory fluids during sleep are not yet well established. **Methods**: This was a small-scale exploratory study conducted on patients exhibiting early symptoms of edema or swelling, or participants who reported discomfort possibly due to such symptoms. Changes in impedance parameters related to inflammatory body fluids were measured in subjects (*n* = 55) lying lay on either a conventional electric mat (non-FIR) or a loess bio-ball mat (FIR) set at 40 °C for 30 min. To assess the effects of heat and FIR exposure during sleep, additional impedance measurements were taken in subjects (*n* = 60) who used either on an electric mat or a loess bio-ball mat set at 30 °C during sleep. **Results**: A total of 115 participants were included in four experimental conditions. In subjects exposed to conductive heat or FIR for 30 min while lying on an electric mat and a loess bio-ball mat set at 40 °C, only minimal changes were observed in impedance parameters and inflammatory fluid-related values. However, significant changes were seen in subjects (*n* = 30) who slept for 7 h on a loess bio-ball mat set at 30 °C. These changes are presumed to results from the deeper sleep and FIR emitted from the loess bio-balls. In contrast, no significant changes were observed in the group (*n* = 30) that used the electric mat at 30 °C under the same sleep conditions. **Conclusions**: Sleeping on a FIR-emitting loess bio-ball mat may stimulate biological tissues and lymphatic circulation, gradually reducing inflammatory fluid accumulation. This suggest potential benefits for improving the physiological condition and function of cell membranes and muscles.

## 1. Introduction

Far infrared (FIR: λ = 4–1000 μm, 12.4 meV–1.7 eV) is a subdivision of the electromagnetic spectrum currently under investigation for its biological effects [1]. Compared to conductive heating, FIR is more energy-efficient and capable of delivering energy deep into the body through radiation [2]. It can penetrate 4–5 cm into the epidermis, providing both thermal and nonthermal effects by increasing the vibrational motion of water molecules within cells and tissues [1]. FIR with wavelengths of 4–16 μm is known to increase tissue temperature through the resonant absorption of energy, thereby enhancing fluid dynamics within tissues [3,4]. Because FIR frequencies overlap with the natural resonance frequencies of water molecules in biological tissues, their effects on human tissues can be amplified via resonance or supposition phenomena [5]. FIR radiation enhances microcirculation in the human body, stimulates cellular growth, and ultimately affects overall metabolic activity [6]. Clinically, FIR is widely utilized in medical institutions and clinical studies [7], where it has been shown to promote blood circulation and improve immune system function [8]. Far-infrared radiation thermotherapy (FIRT) is also recognized as an effective conservative treatment for limb lymphedema, as it increases blood microcirculation and enhances lymphatic circulation in the skin [9].

The use of FIR has expanded across various fields, including housing materials, bedding products, assistive devices, and alternative medical technologies [10,11]. However, the unique FIR-emitting properties of untreated loess are altered or diminished following high-temperature heat treatment. Loess powder heated at 850 °C for 2 h or 1050 °C for 1 h showed a significant reduction in its absorption spectrum, particularly around 9.5–9.8 μm [12]. In contrast, untreated loess powder exhibited strong absorption peaks, primarily attributed to the vibrational motions (e.g., deformation, stretching, and bending) of Si-O bonds, which selectively absorb external IR radiation at similar frequencies. The FIR emitted from loess is likely derived from these vibrational motions of Si-O and exhibit frequencies similar to those of water molecules in the human body. Accordingly, FIR emitted from loess bio-balls can penetrate the epidermis and adipose tissue, transferring radiation energy to water molecules and thereby enhancing their vibrational activity. This process increases core body temperature and activates water molecules in cells and tissues [12]. FIR emitted from loess bio-balls is widely applied for therapeutic purposes in medical settings [13]. It has been reported to reduce inflammation levels not only by increasing core body temperature but also by stimulating cellular activity and enhancing lymphatic circulation, thereby facilitating the removal of waste products [13].

Bioelectrical impedance analysis (BIA) is a rapid and noninvasive technique, widely used to estimate body composition and health-related indicators such as hydration status, malnutrition, disease prognosis, physical fitness, and general health [14,15,16]. This technique offers various bioelectrical parameters by employing equivalent circuit models and mathematical formulas [17,18]. BIA has been applied to diagnose a wide range of diseases in medical settings [19] and is also used to evaluate hydration status, nutritional condition, body composition, muscle-to-fat ratio, degree of obesity, muscle mass balance, and edema [20,21]. Multi-frequency BIA (MF-BIA) has demonstrated accuracy in measuring upper extremity lymphedema in patients by detecting changes in extracellular fluid volume [22,23].

FIR therapy is employed to reduce inflammatory fluids, enhance tissue repair, and promote muscle recovery in various disease conditions [24]. Due to its resonance effects with water molecules, FIR in the wavelength range of 4–16 μm is often referred to as “life rays” [4]. Loess, which emits a significant amount of FIR in the 9.5–9.8 μm, has been widely applied in health-promoting industries, including clothing, natural pigments, health foods, cosmetics, eco-friendly building materials, and biopesticides used to control red algae proliferation in marine ecosystems [25,26].

To evaluate the therapeutic and health-promoting effects of FIR emitted from loess bio-balls that retain the beneficial properties of raw loess, this study measured changes in inflammatory fluid and cell membranes/muscles before and after treatment under various conditions (temperature, time, and sleep). Measurements were conducted in both control (non-FIR) and experimental (FIR) groups.

## 2. Materials and Methods

### 2.1. Bioelectrical Impedance and Equivalent Circuit of Extracellular Fluid (ECF), the Cell Membrane (C_m_), and Intracellular Fluid (ICF)

Bioelectrical impedance (*Z*) is a vector composed of a real component, resistance (*R*), and an imaginary component, capacitive reactance (*Xc*). Resistance is associated with the resistive pathways through body fluids such as extracellular fluid (ECF) and intracellular fluid (ICF), while reactance is related capacitive pathways such as cell membrane structures [27]. Depending on the applied frequency, electric currents follow different paths within biological tissue. At frequencies below 50 kHz, the current (*I*_1_) has low energy and cannot penetrate the cell membrane; thus, it predominantly flows through the ECF (*Re*). At 50 kHz, the current (*I*_2_) becomes strong enough to penetrate the cell membrane, allowing flow through both the ICF (*Zi*) and ECF (*Re*). The impedance values corresponding to current flow through the ECF at low frequencies and through both the ECF and ICF at higher frequencies (above 50 kHz) can be obtained using an MF impedance analyzer (Multiscan5000, Bodystat Ltd., Isle of Man, UK). By applying these impedance values to mathematical models, various physiological parameters such as body composition and total body water (TBW) can be estimated [28]. TBW varies depending on factors such as age, sex, and body mass index (BMI), and constitutes approximately 60% of total body weight. Of this, intracellular fluid (ICF) and extracellular fluid (ECF) account for approximately 40% and 20%, respectively. Additionally, interstitial fluid (ISF), which makes up approximately 15% of ECF and plasma, accounts for approximately 5% of ECF. ISF shares a similar composition with plasma, albeit with lower protein content. Human tissue cells are enclosed by membranes that function like electrical capacitors. Inside the cell lies the ICF, which acts as an electrical conductor, while the external environment consists of ECF containing the ISF [29].

### 2.2. Study Participants

Subjects for the clinical study on inflammation and lymphatic circulation were recruited through an article published in the local newspaper “Yangsan News Park” on 7 November 2023. Additional participants were recruited on 26 December 2024, for further experiments focusing on these conditions during sleep. Eligible participants were adults aged 30 to 80 years who had been diagnosed with inflammation or edema at a medical institution or who reported discomfort due to lymphatic circulation disorders. Participants were volunteers experiencing mild inflammation or swelling, primarily in the early stages of lymphedema—stage 0 (characterized by sensations of swelling, tightness, and a feeling of heaviness without visible external signs) or stage 1 (occasional swelling that subsides when the affected area is elevated) [30]. All participants were non-smokers and had no history of hypertension, asthma, or diabetes mellitus. None were taking medications that could affect autonomic nervous system (ANS) function.

Demographic characteristics and physical condition data for each group are summarized in Table 1. The body water ratio (BWR), defined as the ratio of extracellular fluid (ECF) to total body water (TBW), was used as an indicator of inflammatory status in the participants’ bodies.

### 2.3. Trial Design and Setting

**Figure 1:**
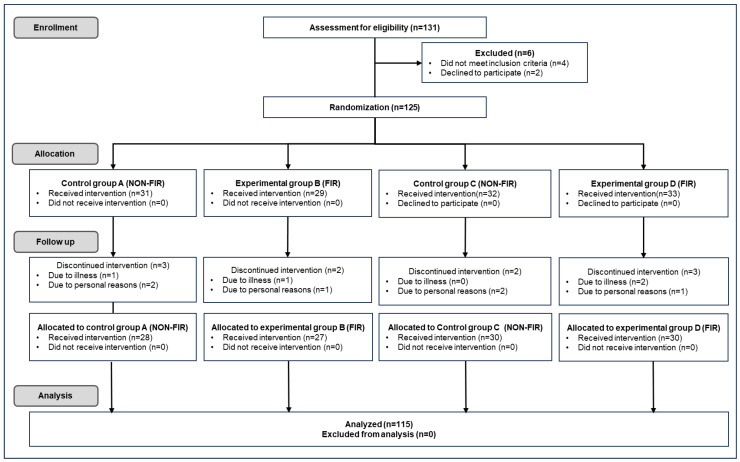
CONSORT diagram illustrating enrollment, group allocation, participation, and experimental data analysis (Adapted with permission from the CONSORT 2010 statement [31]).

The experimental protocol was approved by the Inje University Bioethics Committee (registration number: INJE 2023-05-035-005) on 20 September 2023, as a clinical trial registration titled “Improvement of blood circulation and health promotion effects of related diseases by far-infrared rays emitted from loess bio-balls.” The Inje University Bioethics Committee approved the protocol for additional experiments (registration number: INJE 2023-05-035-007) on 31 December 2024. The study was primarily conducted at the Hanwool Bio Lab and followed the Consolidated Standard of Reporting Trials (CONSORT) guidelines. As shown in Figure 1, the protocol also included self-reported home-based measurements provided by study participants.

The exclusion or discontinuation criteria for the intervention were as follows: pregnant women or applicants with cancer, diabetes, cardiovascular disease, or neurological diseases. Participants with mental weakness or anxiety symptoms, mental illness (including dementia), or those who withdrew owing to illness or personal reasons were excluded from the intervention. Of the 131 recruited participants, 125 participated in the experiment, excluding 4 who did not meet the selection criteria and 2 who dropped out for personal reasons. Participants received a detailed explanation of the experiment and provided written informed consent to participate. Participants (*n* = 60) were first randomly divided into Group A (*n* = 31), who were exposed to heat for 30 min on an electric mat set to 40 °C, and Group B (*n* = 29), who were exposed to FIR on a loess bio-ball mat at 40 °C for 30 min. Another group of participants (*n* = 65) was randomly divided into Group C (*n* = 32), who were exposed to heat for 7 h while sleeping on an electric mat set at 30 °C, and Group D (*n* = 33), who were exposed to FIR for 7 h while sleeping on a loess bio-ball mat set at 30 °C. During the follow-up process for the experiment, three subjects from Group A, two from Group B, two from Group C, and three from Group D were excluded from the experiment owing to illness and personal reasons. After excluding 10 subjects from the experiment, the remaining subjects (*n* = 115) participated in each experiment as groups A (*n* = 28), B (*n* = 27), C (*n* = 30), and D (*n* = 30).

**Figure 2:**
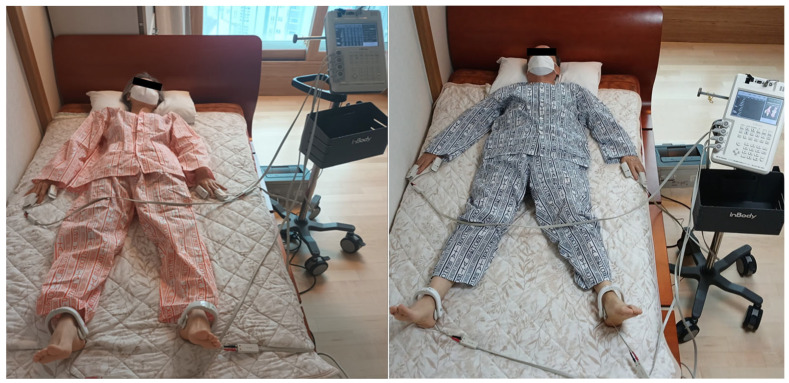
Photograph showing the placement of electrodes for impedance measurement. Clip-type electrodes are attached to both ankles, thumbs, and middle fingers to collect impedance data from segments (both arms, both legs, and trunk).

Impedance measurements to detect changes in inflammatory fluid-related values in the subjects’ trunks were performed using a body water analyzer (InBody S10, InBody, Seoul, Republic of Korea). During the measurement, the participants laid comfortably or slept on an electric mat (SW-301, SHM, Samhwa Electric Blanket, Busan, Republic of Korea), a loess bio-ball mat manufactured by Hanwool Bio, and a Jangsoo bio-ball bed (7111, Jangsoo Industry Co., Ltd., Seoul, Republic of Korea), with electrodes attached to both ankles, thumbs, and middle fingers, as shown in Figure 2.

The effects of conductive heat emitted from an electric mat (non-FIR) and an FIR emitted from a loess bio-ball mat on bioimpedance parameters associated with fluid changes were investigated in this study. First, 28 subjects in Group A (non-FIR) were exposed to conductive heat from an electric mat set at 40 °C for 30 min. There were no changes or minimal changes in bioimpedance parameters or inflammatory body fluids. Second, 27 subjects in group B (FIR) were exposed to FIR emitted from a loess bio-ball mat set at 40 °C for 30 min. Here, there were minimal changes in bioimpedance parameters and inflammatory body fluids. Third, to determine the effects of long-term exposure to conductive heat on bioimpedance parameters, 30 subjects in Group C (non-FIR) were exposed to heat from an electric mat set at 30 °C for 7 h during sleep. Fourth, to determine the effects of long-term exposure to FIR on bioimpedance parameters, 30 subjects in Group D (FIR) were exposed to FIR emitted from a loess bio-ball mat set at 30 °C for 7 h during sleep. Bioimpedance parameters related to body fluid changes were investigated under various conditions (heat/FIR intensity, heat/FIR exposure time, and sleep).

### 2.4. Statistical Analyses

All statistical analyses for control group A (*n* = 28), experimental group B (*n* = 27), control group C (*n* = 30), and experimental group D (*n* = 30) were performed using IBM SPSS Statistics (version 29.0.2.0; IBM Corp., Armonk, NY, USA). A paired sample *t*-test was conducted to compare pre- and post- impedance parameters across different frequencies and groups. Statistical significance was defined as *p* < 0.05. Cohen’s d was used to determine the effect size between two measurements, and was interpreted as follows: d = 0.2 (small effect); d = 0.5 (moderate effect); and d ≥ 0.8 (large effect). Pearson’s correlation coefficient (r) and corresponding *p*-values were calculated to evaluate the relationship among impedance parameters under each condition. Data processing, graph generation, and logistic fitting were conducted using Microsoft Excel 2016 (Microsoft Corp., Redmond, WA, USA).

## 3. Results

### 3.1. Impedance (Z) as a Function of Frequency (f)

Table 2 presents the change in impedance (*Z*) as a function of frequency before and after the application of heat or FIR for four groups subjected to different experimental conditions. ΔZ_E_ presents the change in impedance before and after applying heat on the electric mat, and ΔZ_L_ indicates the change in impedance before and after FIR exposure on the loess bio-ball mat.

For Group A, *Z* is presented as a function of frequency for 28 participants who lay comfortably on a conductive electric mat heated to 40 °C for 30 min. Z_EB_ refers to the impedance measured before heating, while Z_EA_ represents the impedance after 30 min of heating. Overall, impedance decreased with increasing frequency. After heating, a slight increase in impedance was observed compared to pre-heating values. Specifically, measured impedance increased modestly across all frequency ranges, with an average increase of approximately 0.90% to 1.40%. For instance, at 50 kHz, the pre-heating impedance (*Z*_EB_) was 29.16 ± 1.61 [Ω]. After 30 min of heating at 40 °C, the post-heating impedance (*Z*_EA_) increased by 0.30 [Ω] (1.03%), reaching 29.46 ± 1.65 [Ω].

For Group B, *Z* is shown as a function of frequency for 27 participants laying comfortably on a loess bio-ball mat heated to 40 °C for 30 min. *Z*_LB_ refers to the impedance measured before FIR radiation, and *Z*_LA_ indicates the impedance after 30 min of FIR exposure. Similarly to Group A, impedance decreased with increasing frequency. However, following FIR exposure, a slight increase in impedance was observed compared to baseline values. Specifically, impedance increased by approximately 1.74% to 2.52% across the frequency range. For instance, at 50 kHz, the pre-exposure impedance (*Z*_LB_) was 28.64 ± 2.11 [Ω]. After 30 min of FIR exposure at 40 °C, the post-exposure impedance (*Z*_LA_) increased by 0.68 [Ω] (2.37%) to 29.32 ± 2.38 [Ω].

For Group C, Z is presented as a function of frequency for 30 participants who lay comfortably on an electric mat heated to 30 °C for 7 h during sleep. As the frequency increased, impedance decreased, and the measured impedance increased more substantially during sleep compared to the values observed in Groups A and B. After 7 h of heat application during sleep, impedance increased by approximately 4.42 to 5.47% across the frequency range. For example, at 50 kHz, the impedance before heating (*Z*_EB_) was 28.72 ± 2.11 [Ω], whereas after 7 h of heating during sleep, the post-heating impedance (*Z*_EA_) increased by 1.57 [Ω] (5.47%) to 30.29 ± 1.38 [Ω].

For group D, *Z* is presented as a function of frequency for 30 participants who lay comfortably on a loess bio-ball mat heated to 30 °C for 7 h during sleep. As expected, impedance decreased with increasing frequency. However, after 7 h of FIR exposure during sleep, the measured impedance significantly increased compared to pre-exposure values, with an overall increase ranging 4.50% to 8.08% across the frequency range. For instance, at 50 kHz, the impedance before FIR application (*Z*_LB_) was 27.83 ± 1.87 [Ω]. Following FIR exposure during sleep, post-exposure impedance (*Z*_LA_) increased by 2.22 [Ω] (7.98%) to 30.05 ± 2.01 [Ω]. This substantial increase in *Z*_LA_ may be attributed to a reduction in the body’s conductive ions due to prolonged FIR exposure while sleeping on the loess bio-ball mat at 30 °C, or to moisture evaporation from the skin or breath.

Table 3 presents the results of a paired sample *t*-test comparing pre- and post- impedance values across multiple frequency bands (1, 5, 50, 250, and 1000 kHz) for group A to D. Cohen’s d was used to assess the effect size between the two measurements, and is interpreted as follows: d *=* 0.2 (small effect); d = 0.5 (moderate effect); and d ≥ 0.8 or higher (large effect). In Group A, statistically significant differences were observed at 1, 5, 50, 250, and 500 kHz (*p* < 0.05), with effect sizes (Cohen’s d) ranging from 0.63 to 0.90, except for 1000 kHz, where no significant difference was found (*p* = 0.27). In Group B, significant changes were observed across all frequencies except 1000 kHz (*p* = 0.06), with large effect sizes (d = 0.67–1.11). Groups C and D, which underwent sleep-related interventions, showed highly significant differences (*p* < 0.001) across all frequencies. Notably, Group D showed the largest effect sizes, with Cohen’s d values ranging from 0.98 to 1.16, indicating strong treatment effects.

### 3.2. Reactance (X_C_) as a Function of Frequency (f)

Table 4 presents the change in reactance (*Xc*) as a function of frequency before and after the application of heat or FIR for four groups under different experimental conditions. ΔXc_E_ represents the change in reactance before and after applying heat on the electric mat, while ΔXc_L_ denotes the change in reactance before and after FIR exposure on the loess bio-ball mat.

For Group A, the reactance (*Xc*) of the cell membrane is presented as a function of frequency for 28 participants who lay on comfortably on an electric mat at 40 °C for 30 min. *Xc*_EB_ is the reactance measured before heat application, and Xc_EA_ refers to the reactance measured after 30 min of heat exposure. *Xc*_EA_ increased with the frequency range and changed only slightly after heating compared to the pre-heating value (*Xc*_EB_).

For Group B, *Xc* of the cell membrane is shown as a function of frequency for 27 participants who lay comfortably on a loess bio-ball mat heated to 40 °C for 30 min. *Xc*_LB_ denotes the reactance measured before FIR application and Xc_LA_ represents the reactance measured after 30 min of FIR exposure. Xc_LA_ increased across the frequency range and changed only slightly after FIR exposure compared to *Xc*_LB_ before FIR treatment.

For Group C, *Xc* of the cell membrane is presented as a function of frequency for 30 participants lying comfortably on an electric mat heated to 30 °C for 7 h during sleep. *Xc*_EA_ increased with the frequency range and showed a greater increase (1.22–15.83%) compared to the two cases described above. For example, at 50 kHz, *Xc*_EB_ before heat exposure was 2.95 ± 0.60 [Ω], whereas after 7 h of heat exposure during sleep, *Xc*_EA_ increased by 0.19 [Ω] (6.44%) to 3.14 ± 0.56 [Ω].

For Group D, *Xc* of the cell membrane is shown as a function of frequency for 30 participants lying comfortably on a loess bio-ball mat heated to 30 °C for 7 h during sleep. *Xc*_LA_ increased across the frequency range and showed a significantly greater increase (7.14–30.87%) compared to the other three groups. For example, at 50 kHz, *Xc*_LB_ before FIR exposure was 2.85 ± 0.55 [Ω], whereas after 7 h of exposure during sleep, *Xc*_LA_ increased by 0.40 [Ω] (14.04%) to 3.25 ± 0.53 [Ω].

Table 5 summarizes the results of a paired sample *t*-test conducted to compare pre- and post-reactance values across the different frequencies in four control/experimental groups. In Groups A and B, which involved 30 min exposure, no statistically significant changes in reactance were observed, except at 250 kHz in Group A (*p* = 0.03). Compared to Groups A and B, the *p*-values were significantly lower in Groups C and D, which were measured before and after sleep. For instance, in Group C, the *p*-values were below 0.05, with statistically significant differences observed at 50 kHz and 250 kHz (*p* < 0.001). Similarly, in Group D, significant differences were also noted at 5 kHz (*p* = 0.03), 50 kHz (*p* < 0.001), and 250 kHz (*p* = 0.03). These findings suggest that prolonged exposure may lead to more pronounced physiological changes in cell membrane reactance.

### 3.3. Capacitance (C_m_) of Cell Membrane as a Function of Frequency

Table 6 presents the change in capacitance (*C_m_*) of cell membrane as a function of frequency before and after the application of heat or FIR for four groups under different experimental conditions. Δ*C_mEA_* represents the change in capacitance before and after heat application using an electric mat, while Δ*C_mL_*_B_ denotes the change in capacitance before and after FIR exposure using the loess bio-ball mat.

In Group A, the capacitance (*C_m_*) of cell membrane was measured as a function of frequency in 28 participants lying on a conductive electric mat set at 40 °C for 30 min. *C_mEB_* refers to the capacitance measured before heating, and *C_mEA_* is the capacitance measured after 30 min of heat application. The capacitance of the cell membrane exhibited only minimal changes before and after heat exposure. For example, at 50 kHz, the mean *C_mEB_* was 0.97 ± 0.11 [μF], which increased to 0.98 ± 0.13 [μF] after heating—a change of 0.01 [μF] (1.03%).

In Group B, *C_m_* was assessed under FIR exposure in 27 participants lying on a loess bio-ball mat set at 40 °C for 30 min. *C_mLB_* is the capacitance measured before FIR application and *C_mLA_* is that measured after 30 min of FIR exposure. The capacitance values before and after FIR treatment exhibited negligible changes across the frequency range. For instance, at 50 kHz, the *C_mLB_* was 1.08 ± 0.11 [μF], and remained essentially unchanged after FIR application (*C_mL__A_ =* 1.08 ± 0.13 [μF]), indicating no significant effect. In other words, exposure to FIR on a loess bio-ball mat at 40 °C for 30 min had little impact on cell membrane capacitance.

For Group C, *C_m_* is shown as a function of frequency while 30 subjects lay on an electric mat set at 30 °C for 7 h during sleep. The capacitance of the cell membrane decreased relatively significantly over the frequency range compared with the two cases described above. For example, *C_mEA_* at 50 kHz decreased by 0.08 [μF] (7.14%) from 1.12 ± 0.24 [μF] before feat exposure to 1.04 ± 0.19 [μF] after heat exposure. This is believed to be because the inflammatory and waste components of the extracellular fluid around the cell membrane are reduced due to long-term heat exposure and sleep effects [32].

For Group D, *C_m_* was evaluated as a function of frequency in 30 participants who slept on a loess bio-ball mat set at 30 °C for 7 h. The capacitance values were significantly reduced across the frequency range compared with the other three groups. For example, at 50 kHz, *C_m_* decreased by 0.15 [μF] (13.04%), from 1.15 ± 0.21 [μF] before FIR exposure to 1.00 ± 0.15 [μF] after FIR exposure. This reduction is believed to result from a decrease in inflammatory and waste components in the extracellular fluid (ECF) near the cell membrane, likely due to the combined effects of sleep and enhanced lymphatic circulation promoted by FIR emitted from loess bio-balls [11,13,32].

Table 7 presents the results of paired-sample *t*-tests comparing pre- and post- intervention capacitance values across different frequencies and groups. In Groups A and B, no statistically significant differences were observed at any of the measured frequencies, as indicated by high *p*-values (*p* ≥ 0.05) and small effect sizes (Cohen’s d < 0.1). In contrast, Group C demonstrated statistically significant increase in capacitance at both 50 kHz (t = 3.19, *p* < 0.001, d = 0.32) and 250 kHz (t = 3.89, *p* < 0.001, d = 0.51), indicating a moderate effect. Similarly, Group D showed significant increases at 50 kHz (t = 2.27, *p* = 0.03, d = 0.14) and 250 kHz (t = 2.50, *p* = 0.02, d = 0.05), although the effect sizes were smaller.

### 3.4. Resistance (R) Versus Reactance (Xc) Measured at 50 kHz

**Figure 3:**
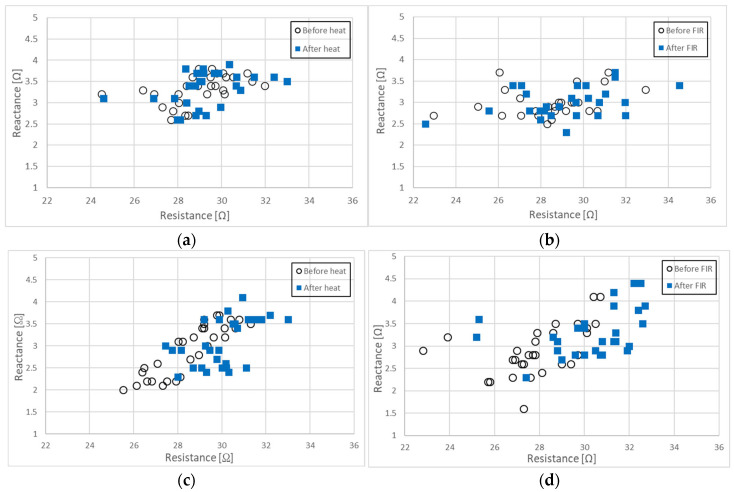
Relationship between *R* and *Xc* measured at 50 kHz: (**a**) before and after 30 min of conductive heating on an electric mat set at 40 °C, (**b**) before and after30 min of exposure to FIR from a loess bio-ball mat set at 40 °C, (**c**) before and after 7 h of conductive heating during sleep on an electric mat set at 30 °C, and (**d**) before and after 7 h of FIR exposure during sleep on a loess bio-ball mat set at 30 °C.

Figure 3 presents a two-dimensional plot illustrating the relationship between resistance (R) and reactance (Xc) at 50 kHz for the four groups under different experimental conditions.

Figure 3a shows the relationship between *R* and *X_C_* measured at 50 kHz for 28 participants before and after 30 min of conductive heating using an electric mat (non-FIR) set to 40 °C. The Pearson correlation coefficients and associated *p*-values for *R* and *Xc* before and after heat exposure were as follows: r = 0.914, *p* ≤ 0.001 for *R*; r = 0.850, *p* ≤ 0.001 for *Xc*. The open circles (○) represent *R* and *Xc* before heat exposure, while the blue squares (□) indicate *R* and *Xc* after heat exposure. There were no statistically significant changes in either *R* or *Xc* following 30 min of conductive heat treatment. The average values of *R* and *X_C_* prior to heat exposure were 29.12 ± 0.75 [Ω] and 3.32 ± 0.35 [Ω], respectively. Following heat exposure, *R* increased by 0.27 [Ω] (0.93%) to 29.39 ± 0.80 [Ω] and *Xc* increased by 0.02 [Ω] (0.30%) to 3.33 ± 0.40 [Ω]. These findings suggest that 30 min of conductive heating at 40 °C has minimal impact on the body fluid distribution or cell membrane characteristics in the subjects.

Figure 3b illustrates the relationship between *R* and *X_C_* measured at 50 kHz before and after exposure to FIR from a loess bio-ball mat at 40 °C for 30 min in 27 participants. The Pearson correlation coefficients and *p*-values for *R* and *Xc* before and after FIR exposure were as follows: r = 0.954, *p* ≤ 0.001 for *R*; *r* = 0.791, *p* ≤ 0.001 for *Xc.* Open circles (○) represent *R* and *Xc* before FIR exposure and blue squares (□) represent *R* and *Xc* after FIR exposure. Minor changes were observed before and after the exposure to FIR. Before FIR exposure, the average values of *R* and *X_C_* were 28.48 ± 2.11 [Ω] and 2.99 ± 0.32 [Ω], respectively. After FIR exposure, *R* increased by 0.72 [Ω] (2.53%) to 29.20 ± 2.41 [Ω] and *Xc* increased by 0.01 [Ω] (0.33%) to 3.00 ± 0.35 [Ω], respectively. These results indicate that when FIR exposure using a loess bio-ball mat at 40 °C for 30 min has minimal impact on body fluids balance and cell membranes characteristics.

Figure 3c presents the relationship between *R* and *Xc* measured at 50 kHz in 30 participants before and after 7 h of conductive heat exposure using an electric mat to 30 °C. The Pearson correlation coefficients and *p*-values between *R* and *Xc* measured before and after heat exposure were as follows: r = 0.909, *p* ≤ 0.001 for *R*; r = 0.879, *p* ≤ 0.001 for *Xc*. Open circles (○) denote *R* and *Xc* before heat exposure and the blue squares (□) represent *R* and *Xc* after heat exposure. Before heat exposure, data points for *R* and *Xc* were primarily distributed in the third quadrant but shifted toward the first quadrant after heat exposure. The mean values of *R* and *Xc* prior to heat exposure were 28.66 ± 1.58 [Ω] and 2.95 ± 0.60 [Ω], respectively. After 7 h of heat exposure, *R* increased by 1.59 [Ω] (5.55%) to 30.25 ± 1.27 [Ω], and *Xc* increased by 0.19 [Ω] (6.44%) to 3.14 ± 0.56 [Ω]. These findings suggest that prolonged conductive heat exposure at 30 °C for 7 h during sleep reduces the inflammatory fluid by 5.55% and improves cell membrane function by 6.44%, potentially due to enhanced skin temperature, respiration, and sleep-related mechanisms. These results align with the observed increase of 5.47% in *Z* and 6.44% in *Xc*, as illustrated in Table 2 and Table 4.

Figure 3d shows the relationship between resistance (*R*) and reactance (*Xc*) measured at 50 kHz before and after exposing 30 participants to FIR emitted from a loess bio-ball mat set at 30 °C for 7 h during sleep. The Pearson correlation coefficients and *p*-values for *R* and *Xc* before and after FIR exposure were as follows: r = 0.909, *p* ≤ 0.001 for *R*; r = 0.879, *p* ≤ 0.001 for *Xc*. Open circles (○) represent *R* and *Xc* before FIR exposure, while blue squares (□) represent *R* and *Xc* after FIR exposure. Prior to FIR exposure, *R* and *Xc* values were predominantly located in the third quadrant and shifted significantly toward the first quadrant following FIR exposure. Before exposure to FIR, the average values of *R* and *Xc* were 27.89 ± 1.88 [Ω] and 2.90 ± 0.57 [Ω], respectively. After 7 h of FIR exposure, *R* increased by 2.35 [Ω] (8.43%) to 30.24 ± 1.96 [Ω], and *Xc* increased by 0.38 [Ω] (13.10%) to 3.28 ± 0.52 [Ω]. These results indicate that FIR exposure from loess bio-balls for 7 h during sleep resulted in an 8.43% reduction in inflammatory fluid and a 13.08% improvement in cellular membrane function. These findings are consistent with the observed increases in *Z* and *Xc* of 7.98% and 14.04%, respectively, as shown in Table 2 and Table 4.

### 3.5. Changes in Body Water Ratio (BWR)

Table 8 presents the changes in body water ratio (BWR), expressed as the ratio of extracellular fluid (ECF) to total body water (TBW), before and after heat and FIR exposure across groups A, B, C, and D. In all groups, a slight decrease in ECF/TBW was observed following the intervention. Group A exhibited a mean decrease in ECF/TBW from 0.383 ± 0.003 to 0.381 ± 0.003, corresponding to a change of −0.002 (−0.522%). Group B showed a reduction from 0.383 ± 0.004 to 0.380 ± 0.004, a difference of −0.00 (−0.783%). In Group C, the ECF/TBW declined more markedly, from 0.381 ± 0.004 to 0.376 ± 0.004, a change of −0.005 (−1.312%). The largest decrease was observed in Group D, from 0.382 ± 0.006 to 0.373 ± 0.009, amounting to −0.009 (−2.356%). Pearson correlation analysis revealed strong positive correlation between pre- and post-exposure BWR values in all groups, with coefficients ranging from r = 0.753 (Group C) to r = 0.918 (Group B). All correlations were statistically significant (*p* ≤ 0.001), indicating consistent individual response to intervention within each group. These findings suggest that both heat and FIR exposure contribute to a modest but statistically significant reduction in extracellular fluid levels relative to total body water, with the extent of change varying slightly among groups.

### 3.6. Muscle Body Water (MBW) Versus Skeletal Muscle Mass (SMM)

**Figure 4:**
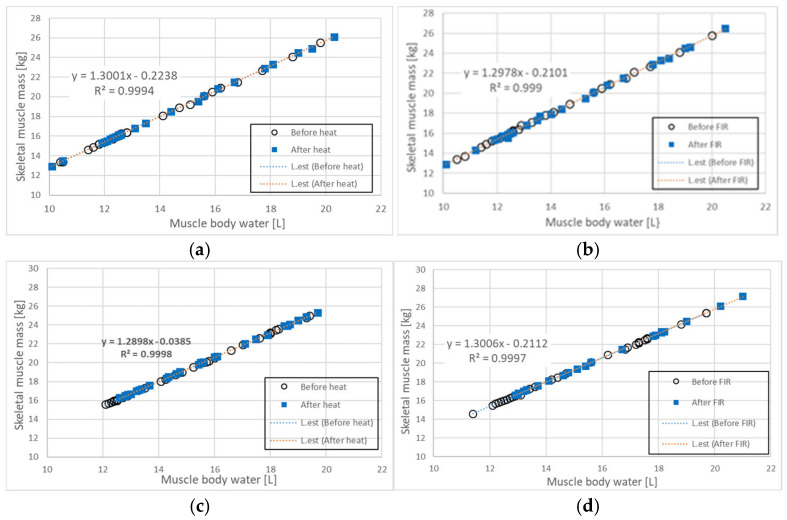
Relationship between MBW and SMM before and after (**a**) 30 min of heat exposure using an electric mat at 40 °C, (**b**) 30 min of FIR exposure using a loess bio-ball mat set at 40 °C, (**c**) 7 h of heat exposure using an electric mat set at 30 °C during sleep, and (**d**) 7 h of FIR exposure using a loess bio-ball mat set at 30 °C during sleep. The blue dotted line represents the estimated linear regression line (L.est) before heat or FIR exposure, while the red dotted line indicates L.est after exposure. All conditions demonstrate strong linear correlations ($R^{2}$ ≥ 0.999), with the most significant changes observed in (**d**) following long-term FIR exposure during sleep.

Figure 4a illustrates the relationship between muscle body water (MBW) and skeletal muscle mass (SMM) in the trunk of 28 participants before and after 30 min of heat exposure using an electric mat set at 40 °C. The Pearson correlation coefficients and *p*-values for MBW and SMM before and after heat exposure were as follows: r = 1.000, *p* ≤ 0.001 (before exposure); r = 1.000, *p*-value ≤ 0.001 (after exposure). Open circles (○) represent MBW and SMM values before heat exposure, while blue squares (□) indicate values after heat exposure. Prior to heat exposure, the mean MBW and SMM values were 14.10 ± 2.72 [L] and 18.09 ± 3.51 [kg], respectively. After heat exposure, MBW increased by 0.35 [L] (2.48%) to 14.45 ± 2.86 [L], and SMM increased by 0.63 [kg] (3.48%) to 18.72 ± 3.70 [kg]. These results suggest that the conductive heat generated by the electric mat had a modest but measurable effect on both MBW and SMM.

Figure 4b shows the relationship between MBW and SMM in the trunk of 27 participants before and after 30 min of FIR exposure using a loess bio-ball mat set at 40 °C. The Pearson correlation coefficients and *p*-values were: r = 1.000, *p* ≤ 0.001 (before FIR exposure), and r = 0.978, *p* ≤ 0.001 (after FIR exposure). As in Figure 4a, open circles (○) represent values before FIR exposure, and blue squares (□) indicate values after exposure. Before FIR exposure, the mean MBW and SMM were 13.90 ± 2.51 [L] and 17.87 ± 3.25 [kg], respectively. After FIR exposure, MBW increased by 0.55 [L] (3.96%) to 14.45 ± 2.83 [L], and SMM increased by 0.67 [kg] (3.75%) to 18.54 ± 3.67 [kg]. These findings indicate that 30 min of FIR exposure via a loess bio-ball mat resulted in a modest increase in both MBW and SMM.

Figure 4c illustrates the relationship between MBW and SMM in the trunk of 30 participants before and after 7 h of heat exposure during sleep using an electric mat set at 30 °C. The Pearson correlation coefficients and *p*-values were: r = 1.000, *p*-value ≤ 0.001 (before exposure), and r = 0.981, *p*-value ≤ 0.001 (after exposure). Open circles (○) represent MBW and SMM values before heat exposure, and blue squares (□) represent values after exposure. Before exposure, the mean MBW and SMM were 15.24 ± 2.48 [L] and 19.59 ± 3.20 [kg], respectively. After heat exposure, MBW increased by 0.42 [L] (2.76%) to 15.66 ± 2.49 [L], and SMM increased by 0.57 [kg] (2.91%) to 20.16 ± 3.20 [kg]. These findings suggest that prolonged low-temperature conductive heat exposure during sleep may positively affect MBW and SMM by enhancing tissue hydration and muscle conditions [32].

Figure 4d shows the relationship between MBW and SMM in the trunk of 30 participants before and after 7 h of FIR exposure during sleep using a loess bio-ball mat set at 30 °C. The Pearson correlation coefficients and *p*-values were: r = 1.000, *p*-value ≤ 0.001 (before FIR exposure), and r = 0.981, *p*-value ≤ 0.001 (after FIR exposure). Open circles (○) indicate MBW and SMM values before FIR exposure, and blue squares (□) represent values after exposure. Prior to FIR exposure, the mean MBW and SMM values were 15.08 ± 2.54 [L] and 19.37 ± 3.30 [kg], respectively. After FIR exposure, MBW increased by 0.93 [L] (6.17%) to 16.01 ± 2.71 [L], and SMM increased by 1.25 [kg] (6.45%) to 20.62 ± 3.53 [kg]. Blue arrows in Figure 4d indicate the range of values before FIR exposure, while red arrows indicate the range after exposure. These results suggest that long-term FIR exposure during sleep may significantly enhance both MBW and SMM through synergistic effects of sleep and FIR therapy [13,32].

## 4. Discussion

For subjects (*n* = 55) exposed to heat and FIR for 30 min using a conductive electric mat or a loess bio-ball mat set at 40 °C, minimal or no changes were observed in the parameters such as *Z*, *Xc*, *C_m_*, *R* vs. *Xc*, ECF/TBW, and the MBW-SMM relationship. However, in the conditions where heat exposure occurred at 30 °C during sleep, noticeable changes attributed to the sleep effect were observed. Particularly, in the subject exposed to FIR emitted from a loess bio-ball mat set at 30 °C for 7 h during sleep, significant changes in these bioelectric impedance parameters were observed, suggesting that both sleep and FIR had synergistic effects.

The substantial increase in impedance (*Z*) by 7.98% at 50 kHz likely reflects water lost due to respiration or transcutaneous evaporation over 7 h FIR exposure during sleep. Conversely, when body fluid and blood components leaked due to peripheral venous infiltration and accumulated in subcutaneous tissue, resistance at 50 kHz significantly decreased by 25.8%, from 498.2 ± 79.3 [Ω] before to 369.4 ± 85.6 [Ω] after infiltration [33]. In support of these findings, previous studies using local bioimpedance analysis in soccer players have reported that *R* values decreased as injury severity increased: 23.1% in grade 3 injuries, 20.6% in grade 2, and 11.9% in grade 1 [34].

Reactance (*Xc*) quantifies the resistance of the cell membrane to an alternating current across a range of frequencies and serves as an indicator of cellular health and integrity [23]. *Xc* also reflects an electrical property derived from the capacitance of the cell membrane and is commonly used to assess cell mass and nutritional status [35]. A 14.04% increase in *Xc* at 50 kHz was observed after 7 h of exposure to FIR using a loess bio-ball mat set at 30 °C during sleep, indicating enhanced cellular health and improved cell membrane function.

When proteins present in inflammatory ECF and metabolic waste products are adsorbed onto the cell membrane, the electrical capacitance (*C_m_*) of the cell membrane increases. Conversely, when these substances detach from the membrane, *C_m_* decreases. In the case of intravenous infiltration assessed using bioimpedance, where blood and infused fluids leak from venous vessels into interstitial fluid (ISF) and surrounding tissues, *C_m_* increased by 28.0% compared to pre-infiltration values [33]. The observed 13.04% decrease in *C_m_* (at 50 kHz) after FIR exposure using loess bio-ball mat during sleep is likely attributable to improved lymphatic circulation induced by both sleep and FIR effects, which reduced the accumulation of inflammatory ECF outside the cell membrane [13].

When muscles are damaged by strenuous exercise, inflammation and swelling can occur, leading to disruption of cell membrane integrity. Local bioimpedance studies assessing muscle damage have reported that resistance values decreased progressively with increasing severity of muscle injury (grade 1: 11.9%; grade 2: 20.6%; grade 3: 23.1%), as did reactance (grade 1: 23.5%; grade 2: 31.6%; grade 3: 45.1%) [34]. In the evaluation of tissue status using bioelectrical impedance analysis (BIA), the injured left arm of patients with exercise-induced muscle damage exhibited higher levels of total body water (TBW), intracellular fluid (ICF), and extracellular fluid (ECF), as well as lower resistance and reactance values, compared to the uninjured right arm, likely due to inflammation and edema [36]. When 30 subjects were exposed to FIR emitted from loess bio-balls for 7 h during sleep, resistance (*R*)—an indicator of inflammatory fluid—was increased by 8.43%, and reactance (*Xc*)—reflecting cell membrane function—increased by 13.10%.

The body water ratio (BWR), defined as the ratio of ECF to TBW, serves as a key indicator of inflammatory fluid retention [35]. The ECF/TBW ratio has been reported to be a more reliable predictor of cancer patient outcomes than other markers, especially in individuals with sarcopenia [37]. In group A (non-FIR), which underwent 30 min of heat exposure using an electric mat set at 40 °C, and group B (FIR), which underwent FIR exposure for 30 min on a loess bio-ball mat set at 40 °C, BWR decreased by 0.522% and 0.783%, respectively. In group C (non-FIR), exposed to 7 h of heat on an electric mat at 30 °C, BWR decreased by 1.312%, indicating the influence of sleep. In contrast, group D (FIR), which was exposed to FIR for 7 h using a loess bio-ball mat set at 30 °C during sleep, showed a significant 2.356% reduction in BWR, attributed to the combined effects of FIR and sleep.

When 30 participants were exposed to conductive heat emitted from an electric mat for 7 h during sleep, MBW and SMM increased by 2.76% and 2.91%, respectively. The increase in MBW and SMM was attributed to the sleep effect. In comparison, when 30 participants were exposed to FIR emitted from a loess bio-ball mat for 7 h during sleep, the increases in MBW and SMM were 6.17% and 6.45%, respectively. These greater increases are attributed not only to the sleep effect but also to the additional influence of prolonged FIR exposure during sleep. Two key mechanisms may explain these results. First, muscle and fluid volume tend to increase during deep sleep. This stage of sleep is associated with heightened growth hormone secretion and enhanced protein synthesis, which contribute to muscle growth and mass accumulation [32]. Rubin et al. [38] reported that the secretion of antidiuretic hormone (ADH) increases during sleep, thereby promoting water reabsorption in the kidneys. ADH secretion is particularly pronounced during deep sleep and is closely linked to sleep architecture. Second, prolonged FIR exposure during sleep may enhance muscle recovery. Chen et al. [11] demonstrated that FIR lamp treatment improved muscle recovery and proprioceptive sensory response following eccentric exercise. Hsieh et al. investigated the application of FIR therapy in soccer-related running injuries and reported favorable outcomes [39]. These findings are consistent with evidence suggesting that FIR-induced sleep, especially of sufficient depth, stimulates vasopressin secretion, enhances renal water reabsorption, and increases body water content. After 7 h of FIR exposure during sleep, MBW and SMM significantly increased by 6.17% and 6.45%, respectively. These results indicate that sleeping on loess bio-ball mats may provide beneficial effects on muscle mass and hydration, contributing to improved sleep quality.

This study has the limitation of being an exploratory preclinical investigation conducted on participants (*n* = 125) who were either diagnosed with early-stage inflammation or edema, or who presented with symptoms suggestive of inflammation at medical institution. Because the study population primarily consisted of individuals in the early stages of the diseases, the generalizability of the findings may be limited. Future studies conducted in a controlled clinical setting involving patients with more advanced stages of inflammation or edema may provide more robust evidence regarding the efficacy of FIR emitted from loess bio-balls in alleviating inflammation, reducing edema, and restoring cell membrane integrity and muscle function.

## 5. Conclusions

The changes in impedance parameters related to inflammatory fluids by the heat from an electric mat and FIR of 9.5–9.8 μm wavelength emitted from a loess bio-ball mat were investigated in participants (*n* = 115) who are in the early stages of inflammation and edema (stage 0 and 1). In an experiment where the subjects were exposed to conductive heat or FIR treatment for 30 min using an electric mat or a loess bio-ball mat set at 40 °C, there were minimal changes in the impedance parameters such as *Z*, *Xc*, *C_m_*, R vs. *Xc*, and ECF/TBW, and the MBW-SMM relationship. Therefore, the changes in impedance parameters related to inflammatory fluids under prolonged heat and FIR exposure were investigated. In the conditions where heat exposure occurred at 30 °C during sleep, noticeable changes attributed to the sleep effect were observed. In particular, in the subject exposed to FIR emitted from a loess bio-ball mat set at 30 °C for 7 h during sleep, significant changes in these bioelectric impedance parameters were observed, suggesting that both sleep and FIR had synergistic effects. In group C (non-FIR), exposed to 7 h of heat on an electric mat at 30 °C, BWR decreased by 1.312%, indicating the influence of sleep. In contrast, group D (FIR), which was exposed to FIR for 7 h using a loess bio-ball mat set at 30 °C during sleep, showed a significant 2.356% reduction in BWR, attributed to the combined effects of FIR and sleep. When 30 participants were exposed to conductive heat from an electric mat for 7 h during sleep, MBW and SMM increased by 2.76% and 2.91%, respectively. The increase in MBW and SMM was attributed to the sleep effect. In comparison, when 30 participants were exposed to FIR emitted from a loess bio-ball mat for 7 h during sleep, the increases in MBW and SMM were of 6.17% and 6.45%, respectively. These greater increases are attributed not only to the sleep effect but also to the additional influence of prolonged FIR exposure during sleep. These results indicate that sleeping on loess bio-ball mats may provide beneficial effects on muscle mass and hydration, contributing to improved sleep quality.

## Figures and Tables

**Table 1:** Demographic characteristics and physical conditions of the study subjects (*n* = 115) in group A, group B, group C, and group D.

Variables	Group A (*n* = 28)	Group B (*n* = 27)	Group C (*n* = 30)	Group D (*n* = 30)
Gender [n (%)]				
Female	12 (42.86%)	10 (37.04%)	16 (53.33%)	15 (51.72%)
Male	16 (57.14%)	17 (62.96%)	14 (46.67%)	14 (48.28%)
Age [years]	63.25 ± 6.05	63.96 ± 5.61	60.27 ± 7.40	59.24 ± 6.82
Height [cm]	168.04 ± 7.81	169.15 ± 7.33	165.12 ± 8.65	164.90 ± 9.38
Mass [kg]	63.66 ± 6.79	63.72 ± 6.91	63.70 ± 9.19	65.41 ± 9.03
BMI [kg/m^2^]	22.59 ± 2.54	22.27 ± 2.44	23.35 ± 2.98	23.99 ± 2.40
BWR	0.385 ± 0.002	0.385 ± 0.004	0.386 ± 0.002	0.387 ± 0.003
Inflammation or swelling	Stage 0, Stage 1	Stage 0, Stage 1	Stage 0, Stage 1	Stage 0, Stage 1

Data are presented as the mean ± standard deviation (SD). Body mass index (BMI) is the value of weight (kg) divided by the square of height (m). Body water ratio (BWR) reflects the inflammatory state of the body as the ratio of extracellular fluid (ECF) to total body water (TBW).

**Table 2:** Impedance as a function of frequency before and after 30 min of exposure to heat or FIR on an electric mat (Group A) and a loess bio-ball mat (Group B), both set at 40 °C. Impedance as a function of frequency before and after 7 h of exposure to heat or FIR during sleep on an electric mat (Group C) and a loess bio-ball mat (Group D), both set at 30 °C.

Group	Frequency [kHz]	1	5	50	250	500	1000
A (n = 28)	Z_EB_ [Ω] at 40 °C, 30 min	34.02 ± 1.80	32.84 ± 1.79	29.16 ± 1.61	25.48 ± 1.44	23.97 ± 1.35	22.14 ± 1.55
	Z_EA_ [Ω] at 40 °C, 30 min	34.34 ± 1.88	33.30 ± 1.86	29.46 ± 1.65	25.83 ± 1.49	24.30 ± 1.45	22.34 ± 1.59
	ΔZ_E_ [Ω] at 40 °C, 30 min	0.32 (0.94%)	0.46 (1.40%)	0.30 (1.03%)	0.35 (1.37%)	0.33 (1.38%)	0.20 (0.90%)
B (n = 27)	Z_LB_ [Ω] at 40 °C, 30 min	33.40 ± 2.34	32.25 ± 2.32	28.64 ± 2.11	25.37 ± 1.94	24.04 ± 1.84	22.45 ± 1.88
	Z_LA_ [Ω] at 40 °C, 30 min	34.15 ± 2.63	32.95 ± 2.53	29.32 ± 2.38	26.01 ± 2.18	24.63 ± 2.07	22.84 ± 2.29
	ΔZ_L_ [Ω] at 40 °C, 30 min	0.75 (2.25%)	0.70 (2.17%)	0.68 (2.37%)	0.64 (2.52%)	0.59 (2.45%)	0.39 (1.74%)
C (n = 30)	Z_EB_ [Ω] at 30 °C, 7 h	33.40 ± 1.99	32.25 ± 2.03	28.72 ± 1.58	25.60 ± 1.12	24.41 ± 0.98	23.30 ± 1.00
	Z_EA_ [Ω] at 30 °C, 7 h	35.09 ± 1.79	33.95 ± 1.75	30.29 ± 1.38	27.00 ± 1.13	25.69 ± 1.09	24.33 ± 1.39
	ΔZ_E_ [Ω] at 30 °C, 7 h	1.69 (5.06%)	1.70 (5.27%)	1.57 (5.47%)	1.40 (5.47%)	1.28 (5.24%)	1.03 (4.42%)
D (n = 30)	Z_LB_ [Ω] at 30 °C, 7 h	32.43 ± 2.33	31.29 ± 2.31	27.83 ± 1.87	24.70 ± 1.55	23.52 ± 1.54	22.21 ± 1.76
	Z_LA_ [Ω] at 40 °C, 7 h	34.86 ± 2.32	33.70 ± 2.31	30.05 ± 2.01	26.58 ± 1.79	25.42 ± 2.54	23.21 ± 2.01
	ΔZ_L_ [Ω] at 40 °C, 7 h	2.43 (7.49%)	2.41 (7.70%)	2.22 (7.98%)	1.88 (7.61%)	1.90 (8.08%)	1.00 (4.50%)

**Table 3:** The results of paired samples *t*-test comparing pre- and post-treatment impedance values across different frequency bands (1, 5, 50, 250, 500, and 1000 kHz) for each group (A–D).

Group	Values	1 kHz	5 kHz	50 kHz	250 kHz	500 kHz	1000 kHz
A (*n* = 28)	t	−2.33	−2.70	−2.33	−2.95	−2.68	−1.13
	*p*-value	0.03 *	0.01 *	0.03 *	0.01 *	0.01 *	0.27
	Cohen’s d	0.74	0.90	0.68	0.63	0.64	0.93
B (*n* = 27)	t	−4.42	−4.23	−4.94	−5.07	−4.56	1.96
	*p*-value	<0.001 **	<0.001 **	<0.001 **	<0.001 **	<0.001 **	0.06
	Cohen’s d	0.88	0.87	0.71	0.67	0.72	1.11
C (*n* = 30)	t	−6.67	−6.75	−6.71	−6.36	−5.77	−3.681
	*p*-value	<0.001 **	<0.001 **	<0.001 **	<0.001 **	<0.001 **	<0.001 **
	Cohen’s d	0.91	0.91	1.09	1.17	1.25	0.65
D (*n* = 30)	t	−14.93	−15.34	−14.31	−12.75	−11.25	−8.26
	*p*-value	<0.001 **	<0.001 **	<0.001 **	<0.001 **	<0.001 **	<0.001 **
	Cohen’s d	0.99	0.99	0.99	0.98	1.00	1.16

Note: *t* = paired samples *t*-test; *p*-value = probability of statistical significance (* *p* < 0.05, ** *p*< 0.01); Cohen’s d = effect size indicator.

**Table 4:** Reactance as a function of frequency before and after exposure to heat or FIR for 30 min on an electric mat (Group A) and a loess bio-ball mat (Group B), both set at 40 °C. Reactance as a function of frequency before and after exposure to heat or FIR for 7 h during sleep on an electric mat (Group C) and a loess bio-ball mat (Group D), both set at 30 °C, respectively.

Group	Frequency [kHz]	5	50	250
A (*n* = 28)	X_CEB_ [Ω] at 40 °C, 30 min	1.78 ± 0.12	3.32 ± 0.35	3.77 ± 0.53
	X_CEA_ [Ω] at 40 °C, 30 min	1.77 ± 0.15	3.33 ± 0.40	3.77 ± 0.58
	ΔX_CE_ [Ω] at 40 °C, 30 min	−0.01 (−0.56%)	0.01 (0.30%)	0.01 (0.00%)
B (*n* = 27)	X_CLB_ [Ω] at 40 °C, 30 min	1.70 ± 0.20	2.99 ± 0.30	3.34 ± 0.54
	X_CLA_ [Ω] at 40 °C, 30 min	1.72 ± 0.24	3.00 ± 0.35	3.39 ± 0.55
	ΔX_CL_ [Ω] at 40 °C, 30 min	0.02 (1.18%)	0.01 (0.33%)	0.05 (1.50%)
C (*n* = 30)	X_CEB_ [Ω] at 30 °C, 7 h	1.64 ± 0.20	2.95 ± 0.60	2.79 ± 0.98
	X_CEA_ [Ω] at 30 °C, 7 h	1.67 ± 0.17	3.14 ± 0.56	3.23 ± 0.90
	ΔX_CE_ [Ω] at 30 °C, 7 h	0.03 (1.83%)	0.19 (6.44%)	0.44 (15.77%)
D (*n* = 30)	Xc_LB_ [Ω] at 30 °C, 7 h	1.68 ± 0.25	2.85 ± 0.55	2.98 ± 0.85
	Xc_LA_ [Ω] at 30 °C, 7 h	1.80 ± 0.22	3.25 ± 0.53	3.90 ± 0.81
	ΔXc_L_ [Ω] at 30 °C, 7 h	0.12 (7.14%)	0.40 (14.04%)	0.92 (30.87%)

**Table 5:** Results of a paired samples *t*-test comparing pre- and post-reactance values across different frequencies (5, 50, and 250 kHz) and control/experimental groups.

Group	Values	5 kHz	50 kHz	250 kHz
A (*n* = 28)	t	−0.46	−0.09	−0.05
	*p*-value	0.65	0.90	0.03 *
	Cohen’s d	0.14	0.22	0.79
B (*n* = 27)	t	−0.38	−0.44	−0.36
	*p*-value	0.70	0.66	0.72
	Cohen’s d	0.25	0.22	0.64
C (*n* = 30)	t	−1.00	−4.63	−3.80
	*p*-value	0.33	*<*0.001 **	*<*0.001 **
	Cohen’s d	0.20	0.33	0.19
D (*n* = 30)	t	−2.23	−3.65	−2.36
	*p*-value	0.03 *	*<*0.001 **	0.03 *
	Cohen’s d	0.25	0.29	0.79

Note: *t* = paired samples *t*-test; *p*-value = probability of significance; * *p* < 0.05, ** *p* < 0.01); Cohen’s d = effect size measure.

**Table 6:** Capacitance as a function of frequency before and after exposure to heat or FIR for 30 min on an electric mat (group A) or a loess bio-ball mat (group B) at 40 °C, respectively. Capacitance as a function of frequency before and after exposure to heat or FIR for 7 h of sleep on an electric mat (group C) or a loess bio-ball mat (group D) at 30 °C, respectively.

Group	Frequency [kHz]	5	50	250
A (*n* = 28)	C_mEB_ [μF] at 40 °C, 30 min	18.25 ± 1.48	0.97 ± 0.11	0.18 ± 0.03
	C_mEA_ [μF] at 40 °C, 30 min	18.24 ± 1.70	0.98 ± 0.13	0.17 ± 0.03
	ΔC_mE_ [μF] at 40 °C, 30 min	−0.01 (−0.05%)	0.01 (1.03%)	−0.01 (−5.56%)
B (*n* = 27)	C_mLB_ [μF] at 40 °C, 30 min	18.97 ± 2.26	1.08 ± 0.11	0.20 ± 0.03
	C_mLA_ [μF] at 40 °C, 30 min	18.98 ± 2.80	1.08 ± 0.13	0.19 ± 0.03
	ΔC_mL_ [μF] at 40 °C, 30 min	0.01 (0.05%)	0.00 (0.00%)	−0.01 (−5.00%)
C (*n* = 30)	C_mEB_ [μF] at 30 °C, 7 h	19.70 ± 2.20	1.12 ± 0.23	0.26 ± 0.11
	C_mEB_ [μF] at 30 °C, 7 h	19.31 ± 2.11	1.05 ± 0.19	0.22 ± 0.08
	ΔC_mE_ [μF] at 40 °C, 7 h	−0.39 (−1.98%)	−0.07 (−6.25%)	−0.04 (−15.38%)
D (*n* = 30)	C_mLB_ [μF] at 30 °C, 7 h	19.35 ± 2.89	1.15 ± 0.21	0.23 ± 0.08
	C_mLA_ [μF] at 40 °C, 7 h	17.99 ± 2.33	1.00 ± 0.15	0.18 ± 0.06
	ΔC_mL_ [μF] at 40 °C, 7 h	−1.36 (−7.03%)	−0.15 (−13.04%)	−0.05 (−21.74%)

**Table 7:** Statistical results of paired samples *t*-test comparing pre- and post-treatment capacitance values across 5 kHz, 50 kHz, and 250 kHz for each group (A–D). The t values, associated *p*-values, and effect sizes (Cohen’s d) are shown below.

Group	Values	5 kHz	50 kHz	250 kHz
A (*n* = 28)	t	0.02	−0.14	0.78
	*p*-value	0.98	0.89	0.44
	Cohen’s d	1.57	0.07	0.03
B (*n* = 27)	t	−0.02	0.02	0.70
	*p*-value	0.99	0.98	0.49
	Cohen’s d	2.54	0.09	0.05
C (*n* = 30)	t	0.85	3.19	3.89
	*p*-value	0.40	<0.001 **	<0.001 **
	Cohen’s d	0.15	0.32	0.51
D (*n* = 30)	t	1.91	2.27	2.50
	*p*-value	0.07	0.03 *	0.02 *
	Cohen’s d	2.72	0.14	0.05

Note: t = paired samples *t*-test; *p*-value = probability of statistical significance (* *p* < 0.05, ** *p* < 0.01); Cohen’s d = effect size measure.

**Table 8:** Changes in body water ratio (BWR) before and after heat or FIR exposure in groups A, B, C, and D. BMR is expressed as the ratio of extracellular fluid (ECF) to total body water (TBW). Subscript B denotes pre- exposure values and subscript A denotes post-exposure values.

Group	ECF/TBW_B_	ECF/TBW_A_	Pearson Correlation Coefficient	Significance Probability
A (*n* = 28)	0.383 ± 0.003	0.381 ± 0.003 (−0.002, −0.522%)	r = 0.816	** *p* ≤ 0.001
B (*n* = 27)	0.383 ± 0.004	0.380 ± 0.004 (−0.003, −0.783%)	r = 0.918	** *p* ≤ 0.001
C (*n* = 30)	0.381 ± 0.004	0.376 ± 0.004 (−0.005, −1.312%)	r = 0.753	** *p* ≤ 0.001
D (*n* = 30)	0.382 ± 0.006	0.373 ± 0.009 (−0.009, −2.356%)	r = 0.888	** *p* ≤ 0.001

Note: r = Pearson’s correlation coefficient; *p* = probability of statistical significance (** *p* < 0.01).

## Data Availability

Data contained sensitive personal and physical information about the study participants and were available from the corresponding author upon reasonable request.

## Abbreviations

The following abbreviations are used in this manuscript:

MBW	Muscle Body Water
SMM	Skeletal Muscle Mass
ECF	Extracellular Water
ICF	Intracellular Water
TBW	Total Body Water
ISF	Interstitial Fluid
MBW	Muscle Body Water
SMM	Skeletal Muscle Mass
ECF	Extracellular Water
ICF	Intracellular Water
TBW	Total Body Water
ISF	Interstitial Fluid
